# Suitability, acceptability, feasibility of modern menstrual methods: a qualitative study in Coimbatore district, Tamil Nadu, India

**DOI:** 10.3389/fgwh.2024.1497686

**Published:** 2024-12-24

**Authors:** P. Aparnavi, Rashmi Ramanathan, Jeevithan Shanmugam, Seetharaman Narayanan, Mohan Kumar, V. Ramya, Ramesh Rathinamoorthy, Sakthivel Vignesh

**Affiliations:** ^1^ Department of Community Medicine, KMCH Institute of Health Sciences and Research, Coimbatore, India; ^2^ Department of Physiology, KMCH Institute of Health Sciences and Research, Coimbatore, India; ^3^ KMCH Institute of Health Sciences and Research, Coimbatore, India

**Keywords:** modern menstrual methods, suitability, acceptability, feasibility, community based participatory research, women, India

## Abstract

**Objective:**

To examine women's perceptions of modern menstrual hygiene methods (MMHM), such as tampons and menstrual cups, focusing on socio-demographic variations and special groups in the Coimbatore district of Tamil Nadu.

**Methods:**

A qualitative study among women of reproductive age (15–49 years) group was conducted using Focus Group Discussions (FGDs) among twelve women subgroups independently in 2023.

**Results:**

The present study involved 23 focus group discussions (FGDs) across various groups of women, including those in formal and informal sectors, urban and rural areas, school and college students, healthcare workers, women in sports, tribal women, transgender women, and female sex workers (FSW), with a total of 188 participants. The age range varied across groups, from 15 to 45 years. Over half of the participants were married (51.1%), and 68.7% were literate, though illiteracy was higher in the informal sector, rural, tribal areas, transgender women, and FSW groups. Sanitary pads were the most used menstrual hygiene method (88.3%), followed by cloth (4.8%), and modern methods like menstrual cups or tampons (1.6%). Notably, 70% of FSW and 28.6% of tribal women still used cloths. Menstrual hygiene choices were often influenced by family recommendations, school-based menstrual hygiene sessions, institutional policies, and social media. Regarding satisfaction, 27.1% were content with their menstrual hygiene method, citing accessibility, affordability, and leakage prevention. However, issues with pads included heat, rashes, and discomfort. A significant number (31.4%) shifted from cloth to pads recently, mainly due to leakage and lack of adequate washing facilities. Only 6.1% had tried modern menstrual methods, with tampons and menstrual cups being considered more suitable but less feasible, especially among tribal women. Participant concerns ranged from the potential health risks of sanitary pads to waste disposal challenges. Recommendations included public menstrual hygiene management (MHM) dispensers, better waste collection practices, and increased awareness through advertisements. Some participants advocated for the concept of free menstruation, emphasizing informed choices and accessibility for all.

**Conclusion:**

The findings suggest that increasing access to modern menstrual hygiene products, coupled with comprehensive education and support, could improve acceptance and feasibility, especially for marginalized and underrepresented women.

## Introduction

Globally, an estimated 1.9 billion women, that is around 23.7% of the population are of menstruating age (1, 2). Women menstruate for about half their lives, with the physical and psychological distress often silenced for ages (3, 4). Despite being a normal physiological process, menstruation has faced challenges in overcoming societal taboos, with growing recognition of its impact on overall quality of life (5). The definition of menstrual health aligns with the WHO definition of health and means access accurate, timely information about menstruation, self-care, and hygiene, along with essential facilities, materials, and health services, ensuring a respectful, stigma-free environment that supports informed decisions and full participation in all aspects of life (5). Menstrual problems vary among different groups, with menstrual hygiene management (MHM) being the most challenging and disproportionately affecting vulnerable populations, including rural, tribal, and transgender individuals. The factors contributing to poor MHM including lack of underwear, products that don't chafe or leak, facilities to wash and change in privacy and with dignity, effective pain management and cultural mores that restrict daily activity together affect the overall health of a women with highest impact in the genitourinary tract (6).

The UNICEF advocates a range of products including menstrual cloths, disposable pads, reusable pads, period panties, menstrual cups, and tampons to be used as menstrual hygiene materials (7). Only about 77.6% of Indian women used hygienic methods of menstrual protection (locally prepared napkins, sanitary napkins, tampons, and menstrual cups) (8, 9). Two percent of women used MMM (tampons and menstrual cups) (10). Ignorance, prejudice, costs, and safety fears can impede girls and women from testing the full range of products available (11).

Modern menstrual methods like tampons and menstrual cups offer significant advantages over sanitary pads, particularly in the Indian context, where menstrual hygiene management often faces challenges due to cultural stigmas, limited access to sanitary products, and inadequate waste disposal systems. Tampons and menstrual cups provide a more discreet, comfortable, and environmentally sustainable option. Unlike pads, which can cause discomfort in hot climates and may lead to rashes due to prolonged contact with moisture, tampons and cups eliminate external contact with menstrual fluid, reducing the risk of skin irritation and infections (10). Menstrual cups, in particular, are reusable, cost-effective over time, and reduce the environmental burden of disposable pad waste, a pressing issue in urban and rural India. Both methods promote better vaginal hygiene by minimizing the risk of bacterial growth associated with prolonged pad use, thus reducing the likelihood of infections that can impact reproductive health. Additionally, by offering longer protection (up to 12 h for menstrual cups), these methods empower women with greater mobility and convenience, encouraging higher participation in education and work without interruptions, ultimately contributing to better overall health and quality of life (12).

Literature evidence informs that menstrual cups and tampons are perceived as satisfactory by women from high-income countries due to better comfort, less leakage, less odor, and less frequent need to change (not tampons) compared to sanitary pads (13, 14). Different studies conducted in low- and middle-income countries have reported the acceptability of menstrual cups among girls and women (15–18). A recent systematic review and meta-analysis on the use, acceptability, safety, and availability of menstrual cups stated that menstrual cups were a safe option for menstruation management (19). However, a gap of context-specific evidence remains with regard to the acceptability and feasibility of using tampons and menstrual cups for menstrual hygiene management among Indian women.

In India, government initiatives and the engagement of non-governmental organizations (for both human and economic resources) to provide menstrual products have increased (20). However, to provide evidence for these policy changes and for women to make informed choices, information is needed on the full range of menstrual products, in particular tampons and menstrual cups, the so-called modern menstrual methods; women's perception including optimism towards, fear and apprehension against modern menstrual methods should be understood.

Against this background, the aim of the present study was to examine women's perceptions of modern menstrual hygiene methods (MMHM), such as tampons and menstrual cups, focusing on socio-demographic variations and special groups in the Coimbatore district of Tamil Nadu. It explored the acceptance, suitability, and feasibility of MMHM among women of reproductive age. Additionally, it investigated women's experiences with their current menstrual hygiene practices, their awareness of available methods, and the factors influencing their choice of menstrual hygiene products.

## Material and methods

### Study area and period

The study was conducted in the Coimbatore district, Tamil Nadu, India. The Coimbatore district, located in the western part of Tamil Nadu, lies about 500 km southwest of Chennai, nestled in the foothills of the Western Ghats. The 2011 census reported a population of 3,458,045 for the Coimbatore district, with 1,728,748 females (50.0%) (21). This study was conducted among women of reproductive age group between July and December 2023.

### Study design

A qualitative study among women of reproductive age (15–49 years) group was conducted using Focus Group Discussions (FGDs) among twelve distinct women subgroups.

### Study population

Only women residing in the Coimbatore district of Tamil Nadu for six months before the date of the survey were included. The study included twelve distinct subgroups categorized based on age, occupation, residence, and unique characteristics. Age-based categories comprised college students and school students, while occupation-based groups included individuals employed in the healthcare sector, those in informal sectors, women farmers, and women employed in the formal sector. Residence-based categories spanned rural, tribal, and urban areas. Special groups of women featured menstruating transgender women, sportswomen, and female sex workers, capturing a broad spectrum of demographic and social contexts.

### Sample size and sampling technique

A minimum of two FGDs among twelve women subgroups accounting for a total of 24 FGDs were planned. However, due to logistical constraints, only one FGD was conducted among transgenders, tribal women, and female sex workers (FSW). The choice of women subgroups and sampling technique are summarized in Table 1. The sampling technique for each group varied due to feasibility with maximum adherence to probability techniques. Briefly, simple random sampling via the random table method was applied to college students, school students, healthcare sector employees, informal sector workers, women employed in the formal sector, and sportswomen. Systematic random sampling of households was used in rural, tribal, and urban areas to select participants based on residence. Snowball sampling was employed to reach women farmers in Vellalore (an urban agglomeration), menstruating transgender women, and female sex workers in Coimbatore, facilitating access to harder-to-reach groups.

**Table 1:** Sociocultural groups included in the study.

Sl. no.	Groups	Sampling technique
Age based categories		
1	College students	SRS (random table method)
2	School students	SRS (random table method)
Occupation based categories		
3	Employed in health care sector	SRS (random table method)
4	Employed in informal sector	SRS (random table method)
5	Women farmers	Snowball technique in Vellalore
6	Women employed in formal sector	SRS technique (random table method) of women in one software company and one industry
Categories based on residence		
7	Rural areas	Systematic random sampling (households)
8	Tribal areas	Systematic random sampling (households
9	Urban areas	Systematic Random sampling (households)
Special groups of women		
10	Menstruating Transgender women	Snowball technique among
11	Sports women	SRS (random table method)
12	Female Sex workers	Snowball technique in Coimbatore

SRS, simple random sampling.

### Data collection tools and procedures

The FGDs were conducted taking guidance from UNICEF's “Focus group discussion guide for communities.” (22) Initially the discussion on the current product use, the problems associated with it, and the unmet need for alternate were conducted. Then a brief IEC regarding MMM (menstrual cups and tampons) was given through audiovisual aids and display of actual products. After explaining the method of use, disposal, prescribed duration of use, and caution and addressing the doubts regarding it the second phase of FGD was conducted. In the second phase based on the information provided women analyzed the suitability, acceptability, and feasibility (SAF) of MMM and reported their perceived SAF and willingness to use the product.

### Variables

The outcome variables assessed were acceptance, suitability, beliefs, apprehensions, challenges, and feasibility of using MMHM.

### Data quality control

The quality of FGD data was ensured through structured protocols, sampling rigor, and thorough data handling procedures. Twelve distinct women subgroups were selected using a combination of probability and non-probability sampling methods, maximizing inclusivity and representativeness. FGDs followed UNICEF's standardized guide, fostering consistency in discussion flow and thematic coverage. Each session was led by the same trained facilitator who adhered to standardized question sets, ensuring uniformity across sessions while allowing flexibility for in-depth responses. The sessions were recorded, transcribed, and verified for accuracy, with transcripts carefully reviewed to minimize interpretation errors. Additionally, regular debriefs and cross-verification among researchers maintained the reliability of findings across FGDs.

### Statistical analysis

For *quantitative data* analysis (data obtained from participants of FGDs), the data obtained from the questionnaire were entered in Microsoft Excel. This master Excel sheet was imported into Software for Statistics and Data Science (Stata) v27 for analysis. Descriptive analyses were presented using numbers and percentages. *Qualitative data* were analyzed using manual, theoretical thematic content analysis following the steps endorsed in Braun and Clarke's six-phase framework (23). The transcripts were read and re-read to ensure familiarity with the data corpus. Also, the notes were made, and early impressions were jotted down. The data were then organized in a systematic meaningful way by generating codes. It was ensured whether the themes made sense, whether data supported these themes, whether trying to fit too much into a theme, whether there were any overlaps, any subthemes within predetermined themes, or other novel themes within the data. The results were presented according to the themes. Under each theme, codes and supportive manually chosen verbatims were provided.

### Ethical considerations

Necessary administrative approvals and clearance from the Institutional Human Ethics Committee (IHEC), KMCH Institute of Health Sciences and Research, Coimbatore, Tamil Nadu were obtained. A team of researchers including the participants in different settings, explained the purpose of the study and invited people to participate. Written informed consent was obtained from all the participants before enrolment into the study.

## Results

In the present study, a total of 23 focus group discussions were conducted—two among women in the formal sector, informal sector, urban, rural, school students, and college students each, three among women in the healthcare sector, women involved in active sports and women in tribal areas, and one among transgender women and FSW. The total number of participants involved in the present study through focus group discussion was 188—with an average of 8.2 participants per FGD. The age group of FGD participants—women in formal sector ranged between 25 and 45 years; women in informal sector between 28 and 42 years; women in urban areas between 18 and 45 years; women in rural areas between 20 and 42 years; school students ranged between 15 and 17 years; college students between 18 and 27 years; women in healthcare sector between 30 and 38 years; women involved in active sports between 18 and 25 years; women in tribal areas between 15 and 44 years; transgender women between 25 and 37 years; and FSW between 32 and 44 years of age. In the present study, a total of 51.1% of participants were married and 68.7% were literate. The rates of illiteracy were higher among women from the informal sector, rural areas, tribal areas, transgender males, and FSW.

### Current choice of menstrual hygiene method

Sanitary pads were the most common choice of menstrual hygiene method in the present study (88.3%, *n* = 166), followed by cloth (4.8%, *n* = 19), and modern menstrual methods (1.6%, *n* = 3) (Table 2). More than two-thirds (70.0%) of FSW and nearly one in three women from tribal areas (28.6%) were currently using cloths. In each focus group discussion, the reasons for the choice of menstrual hygiene method were asked. The majority of the participants cited the recommendation of mothers or any female member in the family during menarche as reasons for choosing the first menstrual hygiene method. School students noted that the menstrual hygiene classes taken as part of the curriculum were useful in choosing or changing the choice of menstrual hygiene method. Similarly, sessions on menstrual hygiene conducted in schools located in the tribal regions of Coimbatore district (for instance, the Government tribal residential primary school) aided the adolescent girls and their parents make informed choices. Institutions also played a role in the choice of menstrual hygiene methods through institutional policies (for instance, mandatory use of sanitary pads by residential students). The role of social media in guiding girls/women towards selecting suitable menstrual hygiene practices should not be understated. Other but important reasons for choosing a particular menstrual hygiene method were lack of suitable environment (for instance, to dry a reusable method or lack of adequate WASH facilities), activities involved in or the type of day-to-day routine (for instance, women involved in active sports required an insertable method, for comfort and to avoid external skin contact/rashes/wetting), cost considerations and individual variations in menstrual flow.

**Table 2:** Verbatims of the thematic analysis on the theme “current choice of menstrual hygiene method”.

Theme	Sub-theme	Verbatims
Reasons for choice of menstrual hygiene method	School students	Verbatim 1.1.1: “*We had classes in our school regarding menstruation, hand washing, and menstrual hygiene; these were special classes taken by our teachers and resource persons”*
	College students	Verbatim 1.2.1: “I used to read and watch videos” Verbatim 1.2.2: “It is an institutional policy in my college to use sanitary pads; cloths are not allowed”
	Urban	Verbatim 1.3.1: “All bathrooms in the house are attached to rooms. The only space where I could sundry menstrual cloths is the balcony. But I am not comfortable with this and so using pads”
	Women involved in active sports	Verbatim 1.4.1: “Tampons and cups are very comfortable while playing; so, I use them for routine. It was recommended by our trainer”
	Tribal women	Verbatim 1.5.1: “The GTRPS (Government tribal residential primary school) conducts sessions on why to use pads, and how to dispose of them. It is given free of cost to us”
	Miscellaneous	Verbatim 1.6.1: “I use pads on the first three days of the cycle. However, the next two days of every cycle I use cloth as pads are costly”
Reasons for change	Pad to cloth	Verbatim 2.1.1: “*During puberty mother gave me a pad but was not comfortable so my mother suggested disposable cloth. I tried cloths and found it to be very comfortable”* Verbatim 2.1.2: “Cloths are my preferred choice. I have low flow, and I am comfortable with menstrual cloths”
	Cloth to pad	Verbatim 2.2.1: “I can throw it off. I don't have to think about washing and drying” Verbatim 2.2.2: “Cloths used to get displaced. I was in fear of leakage while using cloths. I had once stained the bed/car. The stickers available in pads are useful” Verbatim 2.2.3: “I used to get frequent cuts in my thighs while using cloths; change of cloth material did not help” Verbatim 2.2.4: “Cloths smell bad even after washing” Verbatim 2.2.5: “The institute is study has no facilities for washing cloths and drying them in sunlight; so, I had to change to pads” Verbatim 2.2.6: “I had to use pads because I travel frequently; pads being disposable I can throw it” Verbatim 2.2.7: “I am from Ooty. The weather is always cold/rainy and it is difficult for me to dry the cloth”

### Satisfaction, concerns with current menstrual hygiene method, and reported recent change in MHMs

Overall, 27.1% (51 participants) were satisfied with the current choice of menstrual hygiene methods—35.1% reported good accessibility, 28.2% reported affordability, 68.1% reported lack of fear related to leakage and/or displacement, and odor (96.8%). Concerns related to current practices were the need for space to dry (9.6%, with cloths), the heat associated with regular use (18.1%, with sanitary pads), itching (12.2%, with sanitary pads), rashes or cuts on the inner aspect of the thigh (42.0%, with sanitary pads), and pain (3.7%, with sanitary pads).

Nearly one in three participants reported a change from the use of cloths to pads in the recent past (31.4%). The change was common among women from the informal sector (85.7%), urban areas (75.0%), tribal areas (71.4%), formal sector (60.0%), rural areas (50.0%), the health sector (29.4%), and among women involved in active sports (13.8%), in that order. The common reasons were leakage (52.1%), lack of WASH facilities (34.0%), rashes/cuts (13.8%), need to dry in sunlight (13.8%), easy availability at no cost (8.0%, tribal women), and lack of comfort (2.7%). Of the 166 sanitary pad users, 31 users shifted from the use of non-biodegradable to biodegradable pads, with 18 of them citing rashes as the primary reason (58.1%).

### Newer/alternate menstrual hygiene methods tried by the study participants

**Figure 1:**
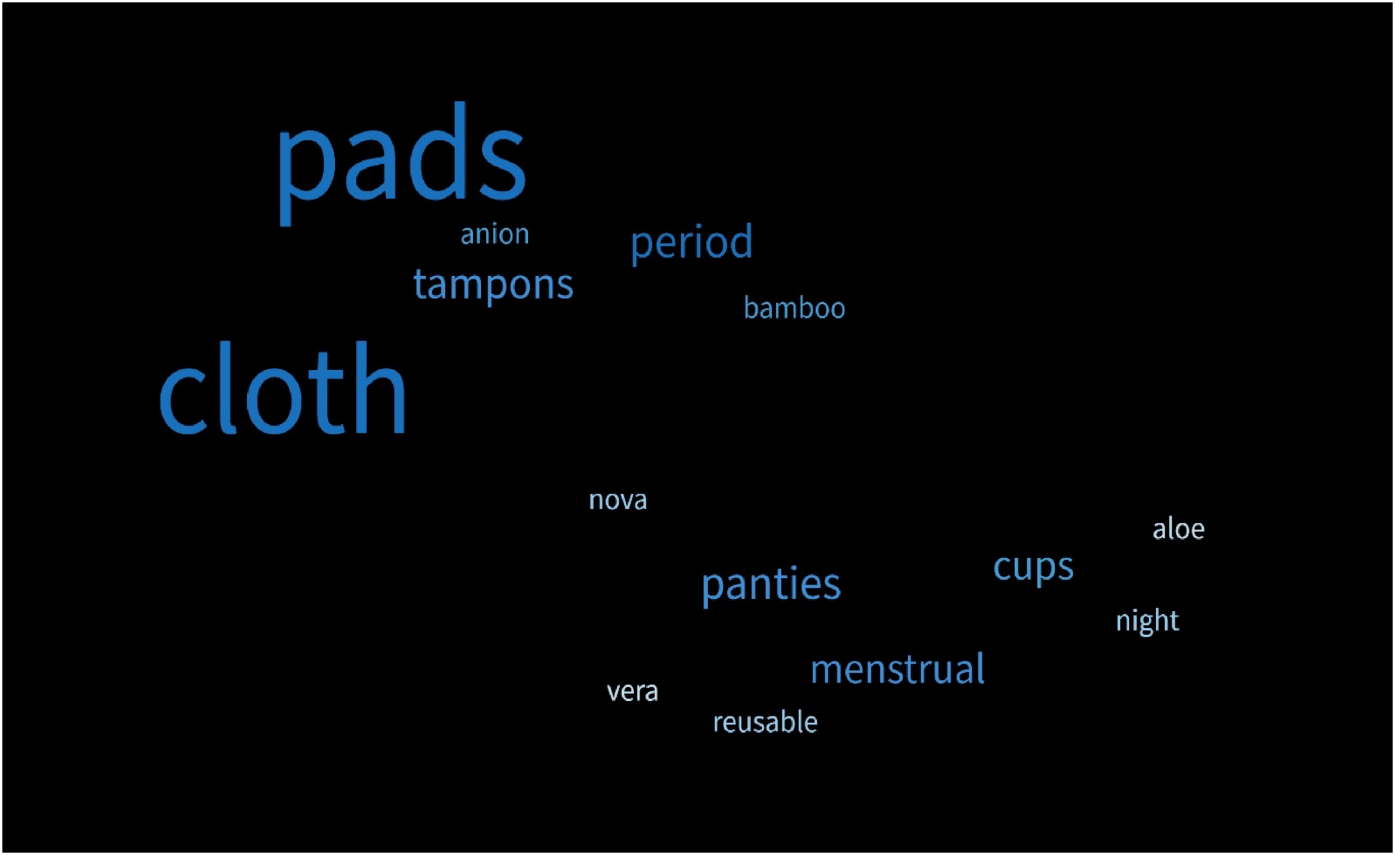
Newer/alternate menstrual hygiene methods tried by the study participants.

In the present study, 48.9% expressed their need for alternate menstrual hygiene methods; of which, 18.0% actively searched for alternate options. More than half the participants (52.1%) were aware of the modern menstrual methods (menstrual cups or tampons)—of which only 6.1% had tried at least one of the modern menstrual methods. The most frequently tried alternate or newer MHMs were cloth pads, period panties, and sanitary pads of Anion or Nova or Aloe Vera or Bamboo types (Figure 1). The participants reported that they knew or heard about the alternate or newer MHMs through YouTube, Instagram, Google ads, doctors, peers/friends, daughters, and daughters-in-law.

The situations that demanded alternative menstrual hygiene methods were during swimming classes, longer duration/distance travel, longer duty hours, and better quality of sleep (Table 3). The reasons for the discontinuation of newer methods tried by the participants were lack of adequate sales points (including only online availability for certain products), frequent stocks, and being expensive. The most common reason for not trying or continuing with any of the modern menstrual methods was fear of insertion into the genital tract (51.1%).

**Table 3:** Verbatims of the thematic analysis on the theme “newer/alternate menstrual hygiene methods”.

Theme	Sub-theme	Verbatims
Newer/alternate MHMs	Reasons for discontinuation	Verbatim 4.1.1: “I tried using organic bamboo pads to escape rashes, but I could not order it at time of need; frequently runs out of stock from supplier” Verbatim 4.1.2: “Anion pads are only available online” Verbatim 4.1.3: “Though I want to use eco-friendly methods, the organic/biodegradable products are very expensive”
	Occasions that necessitated newer/alternate MHMs	Verbatim 4.3: “I have used menstrual cups during my swimming classes. It's very comfortable and there is no leakage”

### Suitability, acceptability, and feasibility

**Figure 2:**
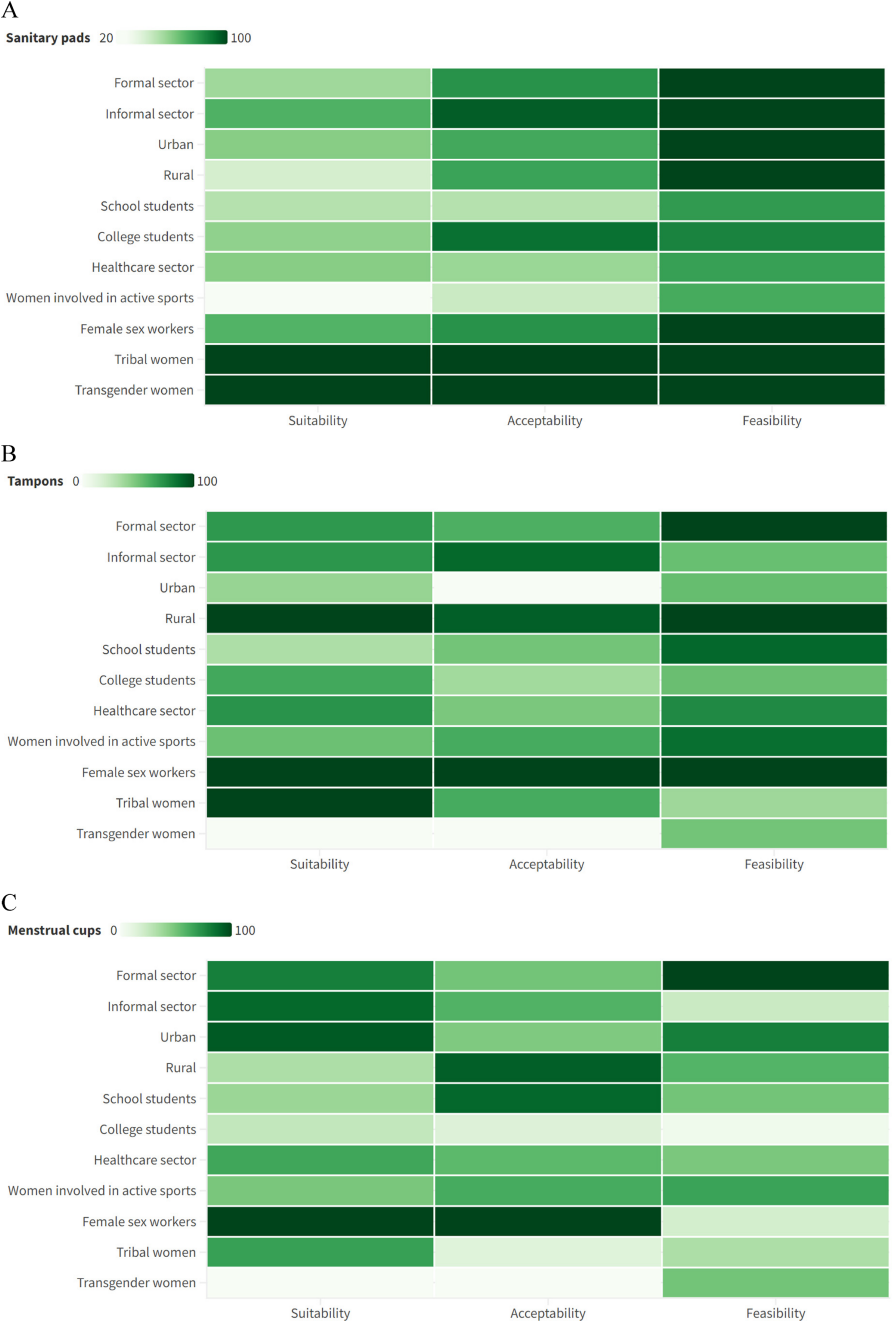
Suitability, acceptability, and feasibility of using **(A)** sanitary pads; **(B)** tampons; **(C)** menstrual cups.

The heat plots showed that overall, tampons and menstrual cups (except for tribal women) had better suitability (Figure 2); and all three methods had equivalent acceptability. However, the feasibility of using menstrual cups was much lower in comparison with sanitary pads and tampons. While the concerns with sanitary pads include leakage, cuts or rashes, and not being user-friendly (particularly during sports); the concerns with MMMs include lack of parent/peer support, inadequate knowledge regarding product/use, and lack of supportive environment (Table 4).

**Table 4:** Verbatims of the thematic analysis on the theme “problems with menstrual hygiene method”.

Theme	Sub-theme	Verbatims
Problems with menstrual hygiene method	Pads	Verbatim 3.1.1: “I don't want to order pads online, and the brand I use is not available near my college; day scholar friends get it for me” Verbatim 3.1.2: “Pads leak even when I am walking” Verbatim 3.1.3: “Every month on the third or fourth day I have rashes with the use of pads. The inside of the thigh region has even darkened; sometimes I notice the sound of pads rubbing with the dress” Verbatim 3.1.4: “While playing pads get soggy and very uncomfortable” Verbatim 3.1.5: “Highly absorbent pads are causing dryness and rashes. But for travel no other choice” Verbatim 3.1.6: “Pads that have blue solution cause more irritation” Verbatim 3.1.7: “Usually during menses, we stay in menstrual huts, take baths in the river, and burn the menstrual product nearby. However, in the rainy season, we have to store the used products and wait for the right time to burn. If we just throw it away it attracts animals” Verbatim 3.1.8: “I have learned to adjust to the discomfort that occurs during the initial days of menstruation. I have changed the way I dress during menstruation days. I understand that rashes will heal on their own. I am used to it.”
	Menstrual cups and tampons	Verbatim 3.2.1: “I wanted to use menstrual cups. Consulted with a doctor, for the same and was told that they are not suitable because I have epilepsy” Verbatim 3.2.2: “My parents were hesitant, so I don't use menstrual cups” Verbatim 3.2.3: “My parents are not allowing me to use insertable products. They are asking me to use the most common product available, that is the pads” Verbatim 3.2.4: “For travel, cups will be difficult to empty and wash My institution lacks adequate facilities in the toilets to clean and wash cups” Verbatim 3.2.5: “I am not sure of the size of the cups. I am hesitant to ask someone regarding this” Verbatim 3.2.6: “I fear cups may fall during change of posture” Verbatim 3.2.7: “I am scared of possible spill during removal” Verbatim 3.2.8: “Use of menstrual cups seems to be time-consuming. I work as a full-time housemaid in XX. I cannot remove the cup, wash it, and reuse it in my workplace. It is not suitable for me. My owner will not like it. I am worried about the cleaning procedure between cycles” Verbatim 3.2.9: “I think tampons might be suitable for me. It is insertable—so no rashes or cuts. And also disposable—so no need to wash” Verbatim 3.2.10: “I am ok using cups at home; but not outside” Verbatim 3.2.11: “In our hostel, sterilizers or other gadgets to clean are not allowed” Verbatim 3.2.12: “I regularly wear sarees. I think while removal of menstrual cups it will spill. It might suit other dresses” Verbatim 3.2.13: “I am nearing menopause, so I won't try new products now. However, I will recommend menstrual cups to my daughter” Verbatim 3.2.14: “During menses, we don't get into our homes. We stay in menstrual huts. How will we be allowed to take washed menstrual products into our homes”

### Participant concerns and recommendations

The concerns or doubts raised by the participants during the FGDs included the adverse effects of using highly absorbent gel-based sanitary pads, the oncogenic potential of sanitary pads/related rashes, the role of MHMs in infertility, the usefulness of flavored sanitary pads, and need for adequate/regular supply of free of cost pads (Table 5). Importantly, concerns were raised on the disposal of sanitary pads in municipality dustbins, associated dog menace, and the handling of these wastes by waste collectors.

**Table 5:** Verbatims of the thematic analysis on the theme “concerns/doubts”.

Theme	Verbatim
Concerns/doubts	Verbatim 4.1.1: “Read newspaper article highly absorbent variety gel is bad for health” Verbatim 4.1.2; “News relating pads with cancer, and infertility are making me anxious” Verbatim 4.1.3: “Flavored pads are effective in combating odor” Verbatim 4.1.4: “Government sister brings pads to our hamlet every month. But not everyone will have a packet. Always it is in short supply. (Tribal)” Verbatim 4.1.5: “When I dispose of the pads in the corporation dustbin, I feel bad for waste collectors who segregate and collect it” Verbatim 4.1.6: “I dispose of the pads using a knotted polythene bag. But street dogs tear off the covers, spread it all over the street creating problems”
Suggestions	Verbatim 4.2.1: “Need a product that can be used along with tight-fitting dresses, light color dresses” Verbatim 4.2.2: “There can be public supply dispenser for menstrual hygiene methods” Verbatim 4.2.3: “Similar to public dispensers, there can be public incinerators at accessible points” Verbatim 4.2.4: “In my municipality, the waste collectors had asked to wrap pads in newspaper and mark it with red ‘X’ mark and dispose separately into waste collector trolley during the day to day collection” Verbatim 4.2.5: “I stay in an apartment. The association has an in-house incinerator where we can dispose of the used sanitary pads” Verbatim 4.2.6: “I think free menstruation should be promoted” Verbatim 4.2.7: “I would suggest that there should be more commercials on tampons and menstrual cups so that it becomes a common product”

The study participants also added their potential solutions to the concerns/difficulties associated with menstrual practices as summarized. It included making available public MHM dispensers, incinerators at private yet accessible points, best practices at the level of waste collection, at residential complexes, and an increase in the number of advertisements so that the newer/alternate methods are known/tried and informed menstrual hygiene choices are made. Even the concept of free menstruation was stressed by the college students.

## Discussion

The current exploratory research covered women across various backgrounds in a series of 23 FGDs. Each FGD spanned a diverse age range, tailored to the respective sectors such as school or college students or rural or urban communities. The prevalence of sanitary pads as the primary MHM method in our study, with 88.3% of participants opting for them, aligns with findings from a previous study conducted in the same district by Durairaj et al. (16), (96.3%) and among Indian women from other regions (24). This consistency underscores the importance of understanding local preferences and access to MHM resources. Though the overall proportion of cloth users was less than 5%, more than two-thirds of the FSW and more than a quarter of tribal women used cloths. The lower prevalence of cloth usage among tribal women may be attributed to the self-reported consistent distribution of government-supplied napkins (20) by their village health workers. Conversely, more than 95% used hygienic methods as defined by NFHS 5, compared to the slightly lower proportion of 77.3% in the NFHS 5 (9). Modern menstrual methods were utilized by 1.6% of women across various sectors, mirroring the 1.4% (16) proportion found in a study exclusively focusing on college-going girls in the same region. However, this comparison might not be entirely valid given the small number of modern menstrual method users in absolute terms.

The diversity in influencers of MHM choices across different populations in the current report highlights the necessity for tailored health education approaches among various sectors. Few participants had changed to a particular method based on their own personal experience or knowledge acquired. A study from Ghana (25) reported that the product of choice was based on individual preference, and experience. The majority of participants cited maternal or familial guidance during menarche as influential in their choice of menstrual hygiene methods. UNICEF (26) acknowledges the discomfort some parents feel discussing periods with their children, highlighting a communication gap. A recent study in the same geographical region (27) revealed that while pre-pubertal talks reduce unmet menstrual needs, less than two-thirds had this opportunity. Hence, despite hesitancy in open discussions, family wields considerable influence on menstrual practices, advocating for open dialogue to promote informed choices. The current study unveiled that menstrual hygiene practices were significantly shaped by the curriculum and influence of schoolteachers. This echoes findings from qualitative research in Ghana (25), where teachers emerged as pivotal sources of information and challenges. Recognizing the crucial role of teachers and the school environment, UNESCO (28) and WHO (29) have spearheaded efforts to integrate comprehensive period and puberty education into school curricular, alongside ensuring the provision of adequate menstrual hygiene management (MHM) facilities in educational institutions. The regulatory approach in health education, known for its immediate impact, was opportunistically employed in promoting menstrual hygiene through institutional policies favoring sanitary pads, showing swift changes from cloth. The current study recorded a significant impact of the service approach of health education with tribal women transitioning from cloth to pads solely due to the doorstep availability of free sanitary pads, as a part of the Jan Aushadhi Suvidha Sanitary Napkins scheme (20). Studies from Ghana and Kenya also supported the service approach, as school attendance and infection control were positively impacted by the free supply of sanitary napkins (30, 31). Social media, was among the influencers of choice of product, and a major source of awareness on MMM in the current report. In line with established evidence (32, 33), social media significantly enhances translational health communication strategies and health literacy (34, 35) by facilitating effective data dissemination. Recognizing its importance, governments worldwide are using platforms like Twitter and YouTube as powerful mass communication tools. However, social media is a double-edged sword to be used with caution and literature has also shown that young people perceive that social media also has a negative role in their life (36). It's important to acknowledge that the lack of facilities also played a role in influencing individuals’ choices of MHM. This was in accordance with the established fact that lower middle-income countries (LMICs), had inadequate access to effective and affordable menstrual products and Water, Sanitation and Hygiene (WASH) facilities.

Approximately half of the participants directly expressed their need for an alternative MHM solution. The insights derived from the suitability of pads currently used by the majority also reveal a concealed felt need among a larger proportion. But only less than one-fifth actively searched for alternate options. This signifies neglect of women's health by thyself. The higher rates of illiteracy among women from the informal sector, rural areas, tribal areas, transgender, and FSW also may contribute to a diminished active pursuit of solutions for health issues. MHM practices globally are influenced by socioeconomic status, personal preferences, local traditions and beliefs, and access to WASH facilities (37). The same was reflected in participants’ decisions regarding the SAF of MMM.

Though used by majority across all the sectors, only tribal and transgender women felt that sanitary pads were suitable for them. With the majority using sanitary pads, this reflects a higher unmet need for menstrual hygiene material. A parallel trend in suitability between tampons and pads with the exception of FSW and rural women lies in their disposability, a shared characteristic. Additionally, their similar cost underscores another point of comparison. Rural dwellers and FSWs in the study shared a commonality: they predominantly spent their time at home, fulfilling roles as homemakers or housemaids. Consequently, the home environment or flexibility in timing their work may be more conducive for tampons or insertable products compared to other groups. Notably, the need to change tampons from time to time due to the risk of toxic shock syndrome that was explained during the IEC might also be the driving factor for considering tampons less suitable. However, this hypothesis is contradicted by the observation of better suitability for menstrual cups, which share a similar risk, among women in sectors where tampons were deemed less suitable. Rural women specifically, considered tampons suitable but not cups because they felt they would not be able to see blood. In contrast to the expectation, younger women, who were primarily engaged in school, college, sports, and healthcare sectors in the current study, found cups unsuitable. This could stem from a common fear of insertable products often observed among unmarried girls (38). Additionally, their work nature might impede timely product changes, or the poor perceived demarcation of feasibility and suitability might influence the results.

The acceptability for the current product, sanitary pads used by many was also poor owing to the problems like rashes, and itching associated with them as shown by other studies from India (38). The acceptability for insertable products, both cups and tampons was high among rural and FSW exhibiting a common pattern with the suitability of tampons. The common factors of age and marital status might positively influence insertable products in these groups. School-going women accepted cups better. Sports women though clearly understood sanitary pads did not suit them, were also apprehensive about the stability and dynamics of insertable products when physically active.

The feasibility of cups was notably low in the current report, and this might primarily be due to the absence of WASH facilities for reusable materials, as reported in a recent study from the same area, which also emphasized high overall unmet needs for reusable products, particularly in the environment domain (27). However, formal sector employees reported a high feasibility for using cups. The school and college sector though having a good WASH facility opined a low feasibility, but in contrast a study from Nepal (39) on school children reported good feasibility. The discrepancy might be in non-users’ apprehensions and users’ experiences or institutional policies as the former majorly had hostel dwellers with policies against the use of electric items or gas which were needed for sterilization of the cups, but the latter was a permitted trial majorly among day scholars. The overall feasibility for tampons was slightly lower than for sanitary pads, which was due to the lack of privacy for an insertable product among construction workers, urban slums, and transgender people reflecting poor WASH facilities. The higher feasibility of tampons in comparison with menstrual cups may be due to their disposable nature. Although schools and colleges typically provide a conducive environment for pad use, unexpectedly, lower feasibility of sanitary pads within the education and healthcare sectors was reported. This could be attributed to a heightened perceived need, such as the desire for incinerators or more frequent emptying of communal pad bins. Additionally, water scarcity issues among the population in this region were highlighted, further contributing to the lower feasibility of the currently used product. With the exception of teenagers, even those who felt that MMM would be suitable and feasible for them to use were not willing to try it due to the apprehension of trying a new product. Neophobia or a ‘negative autosuggestion as to “I don't need; what's the need to change now?’ are established hurdles in bringing about a behavioral change as evidenced by open-air defecation (40) to COVID-19 vaccination (41) and the current report shows that MMM is no exception. The study focused on enabling women to make informed decisions about their MHM.

The present study is not without limitations. The participants who haven't used MMM analyzed the SAF based on the facts provided by the investigators, and this might not reflect the true SAF observed on the use of the product. Only one FGD was conducted due to feasibility concerns among the transgender people and female sex workers who were among the most vulnerable population.

## Conclusion

In conclusion, the present study provides valuable insights into the perceptions, experiences, and challenges faced by women in the Coimbatore district of Tamil Nadu regarding menstrual hygiene methods. Despite growing awareness of MMHM, their adoption remains limited, largely due to cultural taboos, lack of knowledge, and concerns over insertion methods, particularly among special groups like tribal women, transgender individuals, and female sex workers. The overwhelming preference for sanitary pads, despite their reported drawbacks such as rashes and discomfort, highlights the need for targeted education and improved access to safe, affordable menstrual products. The study emphasizes that socio-demographic factors play a significant role in shaping menstrual hygiene practices. Women from urban areas, the formal sector, and younger age groups showed greater openness to newer methods, while traditional practices like using cloths persisted in more marginalized groups, driven by factors like lack of WASH facilities and affordability concerns. Educational institutions and social media were identified as critical influencers in shaping menstrual hygiene choices, particularly among school and college students.

Participant recommendations underline the need for better infrastructure, such as public menstrual hygiene dispensers and incinerators, and increased awareness campaigns to promote safe, informed choices. The findings suggest that increasing access to modern menstrual hygiene products, coupled with comprehensive education and support, could improve acceptance and feasibility, especially for marginalized and underrepresented women. Future interventions should focus on addressing fears and misconceptions about MMHM, fostering supportive environments, and ensuring equity in menstrual hygiene management.

## Data Availability

The raw data supporting the conclusions of this article will be made available by the authors, without undue reservation.

## References

[1] World Health Organization. Women of Reproductive age (15–49 Years) Population (thousands). (2024). Available online at: https://www.who.int/data/gho/indicator-metadata-registry/imr-details/women-of-reproductive-age-(15-49-years)-population-(thousands) (cited September 4, 2024)

[2] UNICEF. Guidance on Menstrual Health and Hygiene. UNICEF (2019). Available online at: https://www.unicef.org/documents/guidance-menstrual-health-and-hygiene (cited September 17, 2024)

[3] Sommer M. Where the education system and women’s bodies collide: the social and health impact of girls’ experiences of menstruation and schooling in Tanzania. J Adolesc. (2010) 33(4):521–9. 10.1016/j.adolescence.2009.03.008 19395018

[4] Mason L Nyothach E Alexander K Odhiambo FO Eleveld A Vulule J ‘We keep it secret so no one should know’–a qualitative study to explore young schoolgirls attitudes and experiences with menstruation in rural Western Kenya. PLoS One. (2013) 8(11):e79132. 10.1371/journal.pone.0079132 24244435 PMC3828248

[5] Hennegan J Winkler IT Bobel C Keiser D Hampton J Larsson G Menstrual health: a definition for policy, practice, and research. Sex Reprod Health Matters. (2021) 29(1):31–8. 10.1080/26410397.2021.1911618 PMC8098749 33910492

[6] Sebert Kuhlmann A Peters Bergquist E Danjoint D Wall LL. Unmet menstrual hygiene needs among low-income women. Obstet Gynecol. (2019) 133(2):238–44. 10.1097/AOG.0000000000003060 30633137

[7] Guidance on Menstrual Health and Hygiene.

[8] Singh A Chakrabarty M Singh S Mohan D Chandra R Chowdhury S. Wealth-based inequality in the exclusive use of hygienic materials during menstruation among young women in urban India. PLoS One. (2022) 17(11):e0277095. 10.1371/journal.pone.0277095 36445854 PMC9707774

[9] National Family Health Survey. Available online at: https://rchiips.org/nfhs/ (cited March 2, 2024)

[10] Wiwanitkit V. Sanitary pad dermatitis. Indian J Dermatol. (2009) 54(4):391. 10.4103/0019-5154.57626 20101351 PMC2807726

[11] Das P Baker KK Dutta A Swain T Sahoo S Das BS Menstrual hygiene practices, WASH access and the risk of urogenital infection in women from Odisha, India. PLoS One. (2015) 10(6):e0130777. 10.1371/journal.pone.0130777 26125184 PMC4488331

[12] Babbar K Garikipati S. What socio-demographic factors support disposable vs. sustainable menstrual choices? Evidence from India’s national family health survey-5. PLoS One. (2023) 18(8):e0290350. 10.1371/journal.pone.0290350 37590271 PMC10434932

[13] Stewart K Greer R Powell M. Women’s experience of using the mooncup. J Obstet Gynaecol. (2010) 30(3):285–7. 10.3109/01443610903572117 20373933

[14] Howard C Rose CL Trouton K Stamm H Marentette D Kirkpatrick N FLOW (finding lasting options for women): multicentre randomized controlled trial comparing tampons with menstrual cups. Can Fam Physician. (2011) 57(6):e208–15. 21673197 PMC3114692

[15] Van Eijk AM Laserson KF Nyothach E Oruko K Omoto J Mason L Use of menstrual cups among school girls: longitudinal observations nested in a randomised controlled feasibility study in rural Western Kenya. Reprod Health. (2018) 15:1. 10.1186/s12978-018-0582-8 30119636 PMC6098596

[16] Durairaj T Aparnavi P Narayanan S Mahantshetti S Dhandapani S Shanmugam J Utilization of modern menstrual methods and related unmet needs among college going women in Coimbatore district: a descriptive cross-sectional study. BMC Womens Health. (2024) 24(1):78. 10.1186/s12905-024-02915-5 38291382 PMC10826201

[17] Beksinska ME Smit J Greener R Todd CS Lee MLT Maphumulo V Acceptability and performance of the menstrual cup in South Africa: a randomized crossover trial comparing the menstrual cup to tampons or sanitary pads. J Womens Health. (2015) 24(2):151–8. 10.1089/jwh.2014.5021 25682816

[18] Averbach S Sahin-Hodoglugil N Musara P Chipato T van der Straten A. Duet for menstrual protection: a feasibility study in Zimbabwe. Contraception. (2009) 79(6):463–8. 10.1016/j.contraception.2008.12.002 19442783

[19] Van Eijk AM Jayasinghe N Zulaika G Mason L Sivakami M Unger HW Exploring menstrual products: a systematic review and meta-analysis of reusable menstrual pads for public health internationally. PLoS One. (2021) 16(9):e0257610. 10.1371/journal.pone.0257610 34559839 PMC8462722

[20] Press Information Bureau. Available online at: https://pib.gov.in/PressReleasePage.aspx?PRID=1983962 (cited December 13, 2023)

[21] Government of India. Census of India 2011, National Population Register & Socio Economic and Caste Census (2011). Available online at: https://censusindia.gov.in/nada/index.php/catalog/42619 (cited November 8, 2024)

[22] Focus group discussion guide for communities.

[23] Peel KL. A Beginner’s Guide to Applied Educational Research using Thematic Analysis. (2020). Available online at: https://openpublishing.library.umass.edu/pare/article/id/1610/ (cited September 17, 2024)

[24] Mathiyalagen P Peramasamy B Vasudevan K Basu M Cherian J Sundar B. A descriptive cross-sectional study on menstrual hygiene and perceived reproductive morbidity among adolescent girls in a union territory, India. J Family Med Prim Care. (2017) 6(2):360. 10.4103/2249-4863.220031 29302548 PMC5749087

[25] Asumah MN Abubakari A Aninanya GA Salisu WJ. Perceived factors influencing menstrual hygiene management among adolescent girls: a qualitative study in the West Gonja municipality of the Savannah region, Ghana. Pan Afr Med J. (2022) 41:146. 10.11604/pamj.2022.41.146.33492 35519170 PMC9046856

[26] UNICEF. Talking About Periods at Home. UNICEF Parenting (2018). Available online at: https://www.unicef.org/parenting/health/talking-about-periods-at-home (cited January 17, 2024)

[27] Ramya V Mohan K Shanmugam J Seetharaman N Mahanshetty S Srihari D Menstrual practice needs among college going women in Coimbatore district, India: an analytical cross-sectional study. Discover Public Health. (2024) 21(1):1–10. 10.1186/s12982-024-00194-x

[28] UNESCO. Menstrual Health and Hygiene Management: A Module for Teachers and Educators. UNESCO (2023). Available online at: https://www.unesco.org/en/articles/menstrual-health-and-hygiene-management-module-teachers-and-educators (cited April 5, 2024)

[29] World Health Organization. Education and Provisions for Adequate Menstrual Hygiene Management at School can Prevent Adverse Health Consequences. (2022). Available online at: https://www.who.int/europe/news/item/27-05-2022-education-and-provisions-for-adequate-menstrual-hygiene-management-at-school-can-prevent-adverse-health-consequences (cited April 5, 2024)

[30] Montgomery P Ryus CR Dolan CS Dopson S Scott LM. Sanitary pad interventions for girls’ education in Ghana: a pilot study. PLoS One. (2012) 7(10):e48274. 10.1371/journal.pone.0048274 23118968 PMC3485220

[31] Phillips-Howard PA Nyothach E Ter Kuile FO Omoto J Wang D Zeh C Menstrual cups and sanitary pads to reduce school attrition, and sexually transmitted and reproductive tract infections: a cluster randomised controlled feasibility study in rural Western Kenya. BMJ open. (2016) 6(11):e013229. 10.1136/bmjopen-2016-013229 27881530 PMC5168542

[32] Ghahramani A de Courten M Prokofieva M. The potential of social media in health promotion beyond creating awareness: an integrative review. BMC Public Health. (2022) 22(1):1–13. 10.1186/s12889-022-14885-0 36544121 PMC9770563

[33] Stellefson M Paige SR Chaney BH Chaney JD. Evolving role of social media in health promotion: updated responsibilities for health education specialists. Int J Environ Res Public Health. (2020) 17(4):1153. 10.3390/ijerph17041153 32059561 PMC7068576

[34] Mythraye VP Kanozia DR. Does media usage enhance nutrition literacy? A systematic literature review and thematic analysis. J Content Community Commun. (2021) 13:328–50. 10.31620/JCCC.06.21/28

[35] Musso M Pinna R Trombin M Carrus PP. Social media to improve health promotion and health literacy for patients engagement. Lect Notes Inform Syst Organ. (2020) 40:103–20. 10.1007/978-3-030-43993-4_10

[36] Mao J Xi FG Jun HJ. The double-edged sword effects of active social media use on loneliness: the roles of interpersonal satisfaction and fear of missing out. Front Psychol. (2023) 14:1108467. 10.3389/fpsyg.2023.1108467/full 36844299 PMC9947851

[37] World Bank. Menstrual Health and Hygiene. (2022). Available online at: https://www.worldbank.org/en/topic/water/brief/menstrual-health-and-hygiene (cited April 6, 2024)

[38] Patel K Dwivedy S Panda N Swain S Pati S Palo SK. Is menstrual cup a sustainable and safe alternative in menstrual hygiene management? A qualitative exploratory study based on user’s experience in India. Clin Epidemiol Glob Health. (2023) 20:101212. 10.1016/j.cegh.2022.101212

[39] Pokhrel D Bhattarai S Emgård M von Schickfus M Forsberg BC Biermann O. Acceptability and feasibility of using vaginal menstrual cups among schoolgirls in rural Nepal: a qualitative pilot study. Reprod Health. (2021) 18(1):1. 10.1186/s12978-020-01036-0 33487171 PMC7831234

[40] Ramesh R SivaRam P. Social & Behaviour Change Communication for Rural Sanitation Professionals Communication Challenges. (2018). Available online at: https://nirdpr.org.in/nird_docs/sb/sb030519.pdf (Accessed December 16, 2024).

[41] Aparnavi P Goswami S Goyal P Singh M Yadav A. Attitude towards uptake of COVID 19 vaccine among pregnant women of rural and urban area of North India. J Comprehens Health. (2022) 10(2):80–4. 10.53553/JCH.v10i02.006

